# Mid- and long-term clinical results of the Elektra and Moovis prosthesis for trapeziometacarpal joint replacement

**DOI:** 10.1186/s12891-024-07439-5

**Published:** 2024-04-25

**Authors:** Pia-Elena Frey, Christin Bühner, Florian Falkner, Leila Harhaus, Benjamin Panzram

**Affiliations:** 1 Department of Orthopedics, University Hospital Heidelberg, Heidelberg, Germany; 2 Department of Hand, Plastic and Reconstructive Surgery, Burn Care Centre, BG Trauma Center Ludwigshafen, Ludwigshafen, Germany

**Keywords:** Trapeziometacarpal joint prosthesis, Thumb carpometacarpal joint prosthesis, Osteoarthritis, Moovis, Elektra, Arthroplasty

## Abstract

**Background:**

Total joint arthroplasty as a surgical treatment option for trapeziometacarpal joint arthritis is recently revived. The aim of this study is to report on mid- and long-term results of the Elektra (single-mobility) and Moovis (dual-mobility) prosthesis for treatment of primary thumb carpometacarpal joint arthritis.

**Methods:**

In this retrospective, monocentric, descriptive cohort study, 31 prostheses were evaluated that were implanted by a single surgeon in 26 patients between 2009 and 2019. Indication for surgery was trapeziometacarpal joint osteoarthritis (Eaton/Littler Stage II and III). Clinical and radiological follow-up was performed at a minimum of 24 months. The postoperative assessment included range of motion, pain, strength as well as functional scores (DASH, MHQ). Implant survival and complications were the primary endpoints.

**Results:**

10 Elektra and 21 Moovis prostheses were implanted between 2009 and 2019 with a mean follow-up of 74.2 months in the Elektra and 41.4 months in the Moovis group. The average patients’ age at surgery was 64 years. Postoperative pain levels (VAS 0–10) were below 2 at rest and under stress in both groups. Grip/pinch strength and range of motion showed results comparable to the contralateral hand. Opposition was excellent with an average Kapandji index of 9.6 in both groups. Elektra achieved slightly better functional scores in the DASH and MHQ score. Satisfaction was high in both groups, and 96% of the patients would recommend the procedure. Metacarpophalangeal hyperextension > 15° was seen in 3 patients per group preoperatively and was corrected to < 5° post-surgery. 3 Elektra prostheses were revised due to cup loosening and dislocation for cup and/or neck replacement or secondary trapeziectomy. 1 Moovis prosthesis was revised with an exchange of the neck to a larger size due to restricted movement. After the mean follow-up of 7.9 years in Elektra and 3.5 years in MOOVIS, cumulative survival was 68.6% vs. 95.2%, respectively.

**Conclusions:**

In this mid- to long-term retrospective analysis, total joint arthroplasty in primary trapeziometacarpal joint arthritis results in low pain levels, excellent mobility and clinical function. Patient satisfaction is overall high. While revision due to cup loosening occurred more often in patients with single-mobility implants, no cases of dislocation or loosening of components were observed in the dual-mobility group.

**Trial registration:**

The study was conducted in accordance with the Declaration of Helsinki, and approved by the Ethics Committee of the Medical Faculty of Heidelberg University, reference number S-150/2020.

## Background

To date, the gold standard of surgical treatment of primary trapeziometacarpal (TMC) joint arthritis remains the resection arthroplasty of the trapezium initially described by Gervis [1]. Once the carpal bone is removed, the remaining options in cases of treatment failure are limited. In comparison to other procedures, the duration of immobilization after trapeziectomy of up to 6 weeks is comparatively long [2, 3].

As an alternative procedure, TMC joint replacement has been established as a trapezium-preserving option: The destroyed joint surfaces are replaced with a ball-and-socket implant design, comparable to the principle well-known from hip arthroplasty. Compared to trapeziectomy, the advantages of arthroplasty are faster and improved restoration of thumb mobility, preservation or restoration of thumb length [4] with improvement of grip and pinch strength, range of motion [3, 5–7] as well as correction of z-deformity [3, 5]. Furthermore, the return-to-work-time is significantly faster [7–9]. While only very limited salvage options are available after trapeziectomy, in case of treatment failure of the prosthesis, a secondary trapeziectomy can be performed with comparably good clinical and functional outcomes regarding primary trapeziectomy [10–12].

In short- and long-term follow-up, numerous models of the first and second generation of TMC joint prostheses with a single-mobility design were associated with various complications – particularly implant dislocation and loosening [12, 13]. In contrast, recent studies reported excellent 10-year survival rates of 95% for the ARPE and 93% for the IVORY prosthesis [13, 14].

The concept of dual mobility was adopted from total hip arthroplasty. It was first introduced in 1969 by Christiansen [15] and further developed by Gilles Bousquet in the early 1970s. Its introduction into the latest generation of TMC joint prostheses in 2012 reduces the shear stress at the cup-bone and metal-PE interfaces and may therefore prevent both PE wear and cup loosening [16, 17]. There are currently three types of these implants on the market (Moovis (Stryker), Touch (KeriMedical) and MAÏA™ (Groupe Lépine; Double Mobility version available since 2005)), of which the Moovis prosthesis was introduced in 2012 and first clinically presented by Schmidt in 2015 [18]. The aim of this study is to report on the mid- and long-term clinical results of the single mobility Elektra and the dual mobility Moovis prosthesis and to extend the available data.

## Methods

In this retrospective, monocentric and descriptive cohort study, 31 prostheses were included that were implanted by a single surgeon in 26 patients between 2009 and 2019 at Heidelberg University Hospital. The indication for surgery was symptomatic primary osteoarthritis of the TMC joint (Eaton/Littler stages II and III) after failure of non-surgical treatment [19]. Surgery was performed via a dorsal approach to the joint and strictly according to the manufacturer’s instructions. The hand was immobilized in a cast for three weeks, after which patients were allowed to perform simple exercises for active mobilization.

All patients that were treated with a carpometacarpal joint prosthesis in our clinic were included in this study if they met the inclusion and exclusion criteria.

Inclusion criteria:

- Age ≥ 18 years
- Consent of the patient to participate in the study

Exclusion criteria:

- Age < 18 years
- Pregnancy
- Language barriers
- Lack of capacity to consent
- Incapability of adherence to the study protocol
- Loss of hand function due to the following comorbidities and previous surgeries: replantation, plexus or nerve damage, arthrodesis or involvement of other fingers.

Clinical and radiological follow-up was performed at a minimum of 24 months. After identification of eligible patients, they were contacted by letter and invited to participate in the study. If the patients agreed, they were given an appointment at the outpatient clinic for the follow-up examination, which was performed by the same person. Postoperative assessment included active range of motion, pain (VAS 0–10), strength (JAMAR dynamometer, pinch gauge), and functional scores (DASH, MHQ). Satisfaction with outcome was rated from 1 to 5: 1 = very dissatisfied, 2 = dissatisfied, 3 = neutral, 4 = satisfied, 5 = very satisfied, and patients were asked if they would recommend the procedure or undergo surgery again. Implant survival and assessment of adverse events were the primary endpoints. Preoperative x-rays were analyzed to determine hyperextension deformity in the metacarpophalangeal joint of the thumb (MCP 1) as well as the stage of osteoarthritis according to Eaton and Littler. The postoperative radiographic imaging of Kapandji posterior-anterior (PA) and lateral views was analyzed for residual hyperextension as well as signs of heterotopic ossifications, radiolucency, implant dislocation and loosening. The radiographs were analyzed by a surgeon who was not involved in treating the patients and by an independent radiologist.

From 2009 to 2014, the Elektra Single-Mobility Prosthesis (SBi, New York, United States) was implanted, before the brand was acquired by Stryker in 2014 with development of the Moovis dual-mobility prosthesis (Stryker, Kalamazoo, Michigan, United States). The cementless Elektra prosthesis with a metal-on-metal tribological pairing consists of a metacarpal titanium stem (4 sizes) and a conical chromium-cobalt cup which are both coated with hydroxyapatite [20].

The Moovis prosthesis is a modular implant with dual mobility. The cementless titanium metacarpal stem with hydroxyapatite coating is available in anatomical (small, medium and large) and non-anatomical (extra small, small, medium and large) designs. The cup is made of cobalt-chromium, coated with titanium and hydroxyapatite and is available in three diameters and versions (8 mm cemented, 9 mm press-fit, 10 mm threaded). The steel neck is available in 3 sizes. The polyethylene head articulates with the metal cup according to a PE-on-metal tribological pairing. In this study, the non-cemented press-fit cups were implanted depending on the trapezium height, bone quality and intraoperative findings. All patients received the standard (non-anatomical) stem component.

### Statistics

Descriptive statistics were applied to analyze the results. When not stated otherwise, mean and 95% confidence interval (CI) or range have been calculated. Kaplan-Meier method was used for survival analysis with revision surgery as the endpoint. Statistical significance was assumed at *p* < 0.05. All values were calculated with IBM SPSS Statistics (Version 26).

## Results

A total of 31 implants (10 Elektra and 21 Moovis) that were implanted in 26 patients between 2009 and 2019 could be included in this study. In 5 patients, surgery was performed on both hands.

### Patient characteristics

The average age at surgery was 64 years (range: 49–80 years), and most of the patients were women with 80% female patients in the Elektra group and 67% female patients in the Moovis group. In 50%, the dominant hand was affected. The mean follow-up was 94.2 (range: 17.9-133.3) months in the Elektra and 41.4 (range: 25.3–69.0) months in the Moovis group with a minimum follow-up of 24 months.

### Survival

**Fig. 1:**
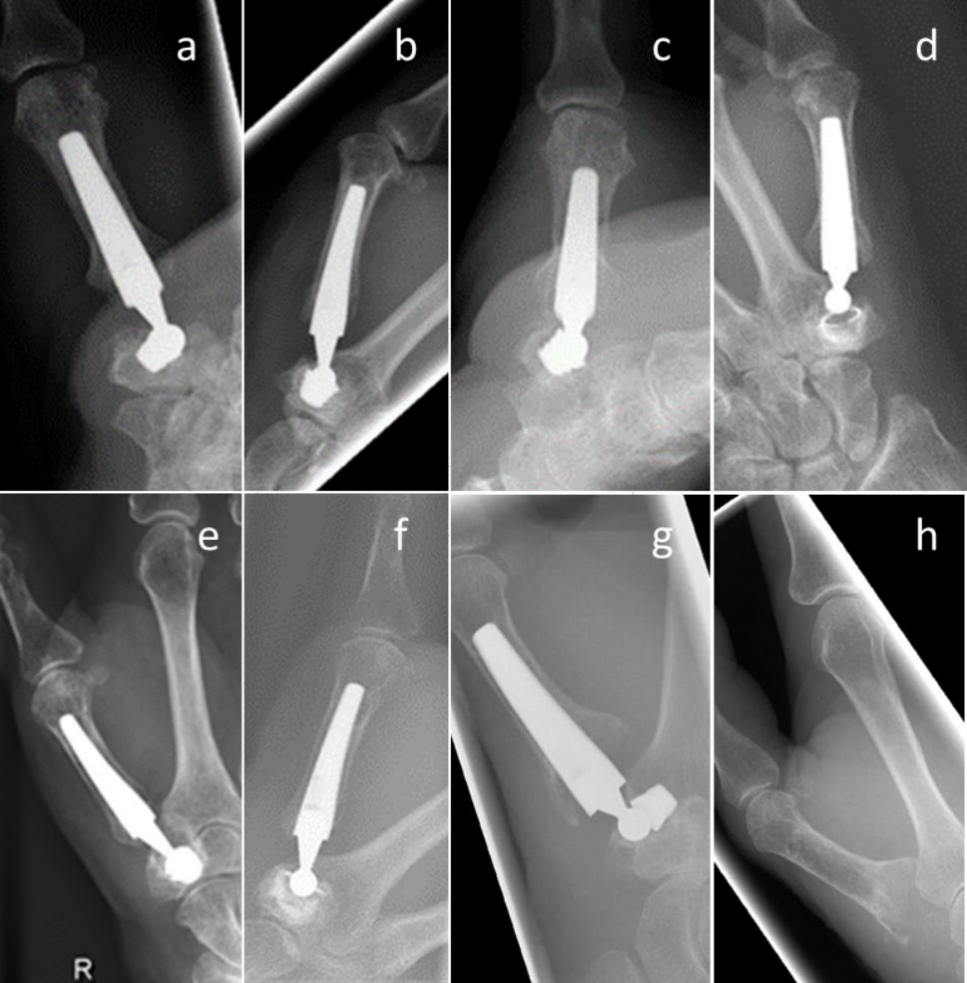
Radiographic imaging of adverse events in 3 patients with an Elektra prosthesis before and after revision surgery. Patient 1: (**a**) Cup loosening, treated with replacement of the cup and neck component (**b**) 6 months after primary surgery. (**c**) Second cup loosening in the same patient treated with replacement of the cup and neck component (**d**) 51 months after primary surgery. Patient 2: (**e**) Cup loosening treated with replacement of the cup and neck component (**f**) 84 months after primary surgery. Patient 3: (**g**) Cup loosening treated with secondary trapeziectomy (**h**) 18 months after primary surgery

Implant failure was defined as any adverse event leading to revision surgery with the exchange of at least one implant component, or secondary trapeziectomy. Prior to revision surgery, CT imaging was performed to identify associated bone pathology. 1 patient in the Elektra group had evidence of scaphotrapeziotrapezoid (STT) arthritis on CT scans, which did not correlate with clinical symptoms. In 3 out of 10 cases, the Elektra prostheses were revised due to cup loosening or dislocation (Fig. 1).

**Fig. 2:**
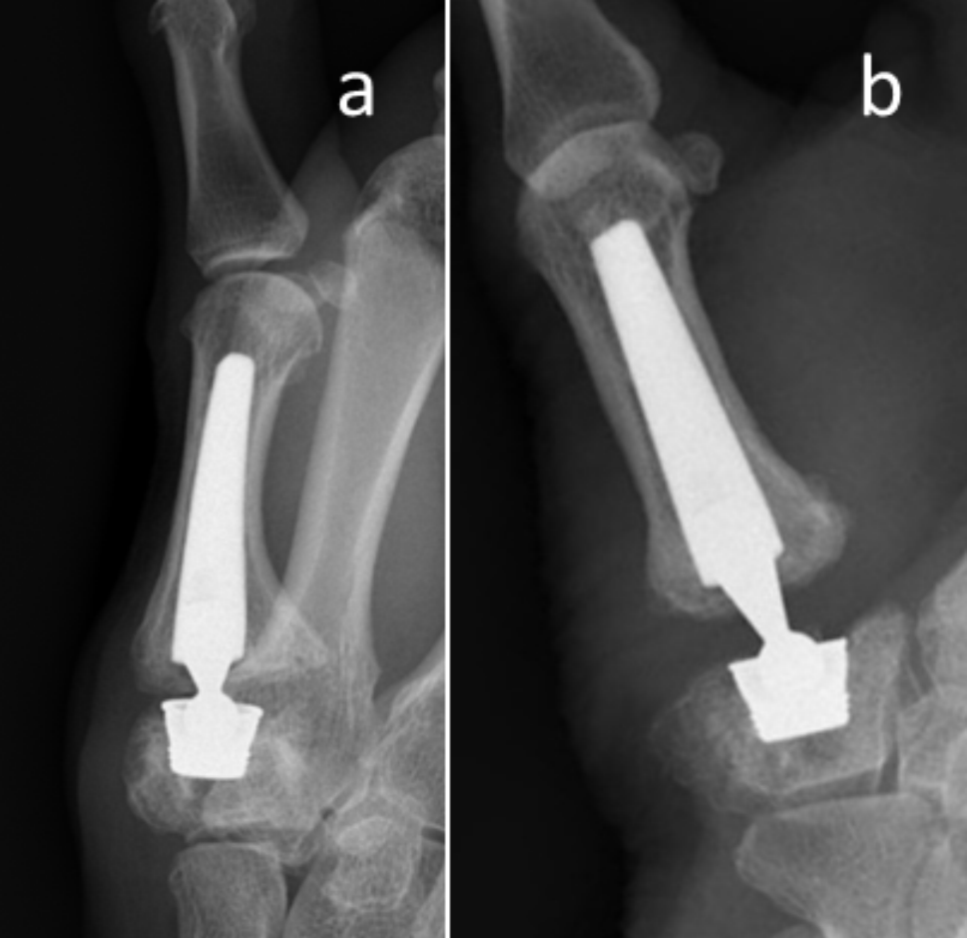
Radiographic imaging of adverse events in 1 patient with a MOOVIS prosthesis before and after revision surgery. Patient 1: (**a**) Restricted movement, treated with exchange of the neck component to a larger size (**b**) 15 months after primary surgery

In the Moovis group, 1 of the 21 prostheses was revised (Fig. 2). Two reoperations were performed due to de Quervains disease after 5 and 9 months, respectively, as well as the removal of ossifications after 32 months due to impingement. No cases of infection were reported in both groups.

**Fig. 3:**
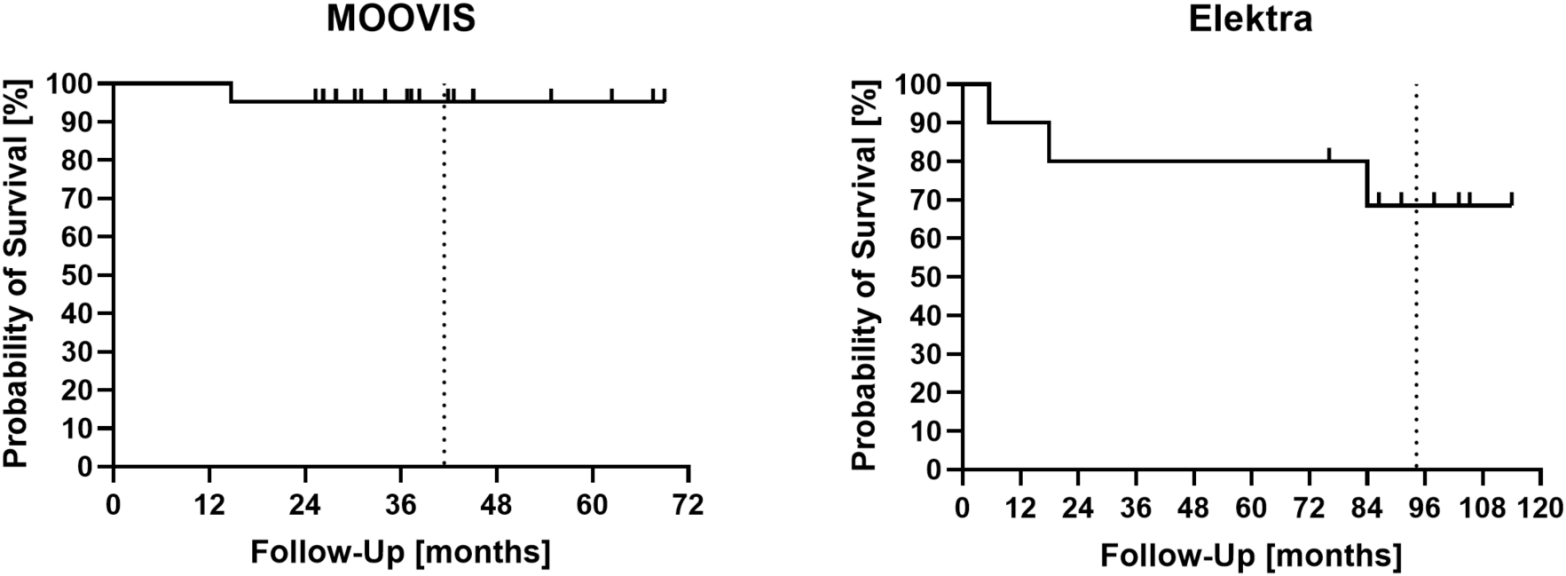
Survival of MOOVIS and Elektra prosthesis as interpreted with the Kaplan-Meier analysis. The dotted vertical line represents the mean follow-up of 41.4 months in the MOOVIS and 94.2 months in the Elektra group

After the mean follow-up of 7.9 years in Elektra and 3.5 years in Moovis, cumulative survival was 68.6% (95% CI: 30.5–88.6) and 95.2% (95% CI: 70.7–99.3), respectively (Fig. 3).

### Functional results

Pain levels (VAS 0–10) were below 2 at rest and under stress in both groups. Grip/pinch strength and active range of motion showed results comparable to the contralateral, not affected hand. Opposition was excellent with an average Kapandji score of 9.6 in both groups. Elektra achieved slightly better functional scores in the DASH and MHQ scores. Most patients were satisfied or highly satisfied in both groups, and 96% of the patients would recommend the procedure. A comparison to preoperative data was not possible as these were not collected prior to the intervention. Patients with bilateral arthroplasty were excluded from the comparative analysis to the contralateral hand. The results are summarized in Table 1.

**Table 1:** Summary of the clinical results of the Moovis and the Elektra group

Criteria [mean (95% CI)]	Implant			
	Moovis		Elektra	
	Operated	Contralat.	Operated	Contralat.
Pain at rest, VAS 0–10	1.3 (0.5–2.1)		0.9 (-0.7-2.5)	
Pain under stress, VAS 0–10	1.9 (1.0–2.8)		1.9 (-0.1-3.9)	
Grip strength, kg	19.0 (14.4–23.5)	17.0 (9.1–24.9)	18.9 (11.0–26.8)	19.1 (10.6–27.6)
Pinch strength, kg	3.4 (3.1–3.7)	3.2 (2.7–3.6)	3.6 (3.0–4.1)	3.4 (2.4–4.4)
Ex/Flex MCP1 joint	47.5° (37.8–57.3)	47.0° (31.9–62.1)	58.7° (48.6–68.8)	49.7° (35.9–63.5)
Active radial abduction	52.2° (48.6–55.7)	53.1° (43.8–62.4)	53.6° (47.5–59.6)	55.8° (43.2–68.4)
Active palmar abduction	47.5° (44.5–50.6)	48.0° (41.4–54.6)	45.9° (41.8–50.0)	48.7° (39.2–58.1)
Kapandji opposition	9.6 (9.2–10.0)		9.6 (8.9–10.2)	
Patient satisfaction, 1–5	4.3 (3.9–4.7)		4.9 (4.6–5.1)	
DASH	25.9 (16.2–35.6)		20.6 (8.5–32.7)	
MHQ	80.2 (74.0–86.3)		85.0 (73.7–96.2)	

Mean and 95% CI are shown below. Whenever available, the results of the treated and the contralateral, not affected side are listed. Statistical comparison between the treated and the respective contralateral hand could not find a significant difference in any of the examined parameters (*p* > 0.05)

### Radiological results

**Fig. 4:**
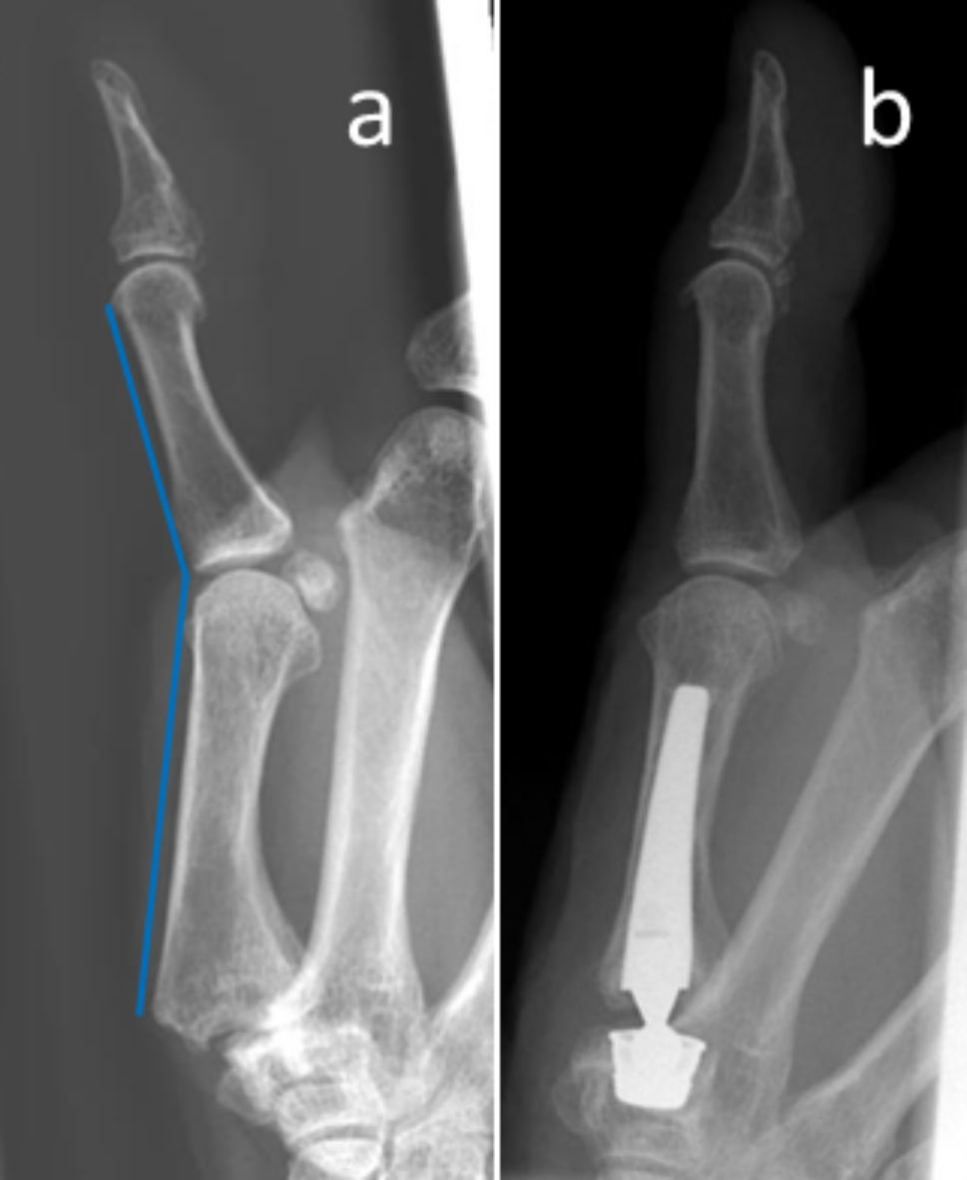
Example of a hyperextension deformity > 15° in a patient with trapeziometacarpal joint arthritis before surgery. After the intervention, the deformity receded completely. (**a**) Before surgery, (**b**) after arthroplasty

Analysis of preoperative radiographs showed that all patients had moderate to advanced stages of osteoarthritis, and approximately 90% in both groups were classified as Eaton and Littler stage III. Signs of STT osteoarthritis were seen in 1 patient preoperatively and 2 patients at follow-up, none of whom were symptomatic at clinical evaluation. Metacarpophalangeal hyperextension of > 15° (range: 15–26°, mean: 23°) was seen in 3 patients per group and could be corrected to 0–5° in all cases (Fig. 4).

The postoperative x-rays at follow-up revealed that none of the Moovis implants had signs of relevant loosening of the components. In addition, heterotopic ossifications were seen in 1 patient with consecutive revision (removal of the ossifications) due to associated pain.

In the Elektra group, radiological alterations were seen in 4 of 10 cases. In 1 prosthesis, a minimal sintering of the stem was seen in comparison to the immediate postoperative imaging, but there were no signs of osteolysis or loosening at follow-up. In 1 patient (after their second revision surgery), protrusion of the prosthesis head beyond the rim of the cup is visible. The 3 other adverse events were previously described in the paragraph *Survival.*

## Discussion

In this retrospective, monocentric, descriptive cohort study, we evaluated the functional and radiological results, implant survival and adverse events of 10 single-mobility and 21 dual-mobility prostheses. Patients were eligible for arthroplasty if they had symptoms of TMC arthritis and non-surgical treatment had failed. All were classified as Eaton and Littler stage II or III according to preoperative radiographs. Newer classifications have now been introduced and it should be emphasized that the choice of treatment was and should be based on the combined assessment of history, clinical examination, functional demands and radiographic evaluation [21, 22]. In this study, the dorsal approach to the TMC joint was chosen. A study comparing different approaches to trapeziectomy suggested that the anterior approach may have comparable or better outcomes than the posterior incision [23].

With an implant survival of 95.2% in the Moovis cohort after the mean follow-up of 3.5 years and 68.6% after the mean follow-up of 7.9 years in the Elektra cohort, our findings are in accordance with the results of recent literature. In the systematic review of Holme et al. published in 2021, failure rates with a range of 2.6–19.9% for different implant types were reported, with 19.9% failure rate after a mean follow-up of 48 months for the Elektra prosthesis [17]. Consistently, we found a survival rate of 80% after 48 months in our Elektra group due to revision after cup loosening or dislocation. In total, 3 of 10 prosthesis were revised after the occurrence of implant failure of the cup component. In one patient, a protrusion of the prosthesis head was seen after the second revision. To date, this patient has not experienced any clinical symptoms such as pain, instability or reduced range of motion and therefore further revision was not recommended. In the revision of the patient treated with a secondary trapeziectomy, the implant was completely removed, including the stem. It should be emphasized that complete osseointegration of the stem is common and therefore not removed as a standard unless it protrudes beyond the resection plane at the base of the first metacarpal or signs of infection are detected.

In comparison, the failure rate of the Elektra prosthesis is among the highest of all of the available implant designs [17]. Possible reasons for this could be the single mobility design combined with the metal-on-metal bearing and threaded cup as opposed to the metal-on-PE tribological pairing used in the MOOVIS implant [20].

Due to the high incidence of adverse events, the Elektra implant was eventually withdrawn from the market. The metal-on-metal bearing may not only contribute to the formation of pseudotumours and loosening in response to metallosis, but may also increase blood metal ion levels [24]. In general, wear debris of the components after TMC arthroplasty is associated with implant loosening (7.1%), osteolysis (1.2%) and metallosis (0.6%) [25], and pronounced polyethylene damage can also occur with dual-mobility prostheses [24].

Lussiez et al., who extensively studied the new-generation, dual-mobility Touch (KeriMedical) trapeziometacarpal prosthesis, postulate a better fixation of the implants in the bone as well as a higher overall stability due to the duo-mobile design, and emphasize that radiolucency is not necessarily a predictor of loosening [26]. Other aspects that significantly influence the risk of dislocation include cup positioning in cases with normal or abnormal trapezium anatomy and iatrogenic aspects: It has been established that the cup component should be positioned parallel to the proximal and distal articular surfaces of the trapezium with an angulation of 7° of flexion relative to the proximal surface in the lateral view [27, 28]. In cases of trapezium dysplasia, the cup should be aligned with the proximal articular surface and using the central axis through the scaphoid in the PA view as a guide [29]. Iatrogenic factors include the choice of head-neck components that are too short. Stability should be determined by intra-operative dynamic fluoroscopy and can be assumed if the head does not protrude beyond the rim of the cup [30]. A recent study by Druel et al. [31] on the long-term results of the ARPE® prosthesis concluded that most adverse events, including implant dislocation, occurred within the first 5 years after implantation. The authors postulate that this may be due to both the single mobility design and lack of experience and technical errors by surgeons as part of the learning curve, which is a known factor to consider in trapeziometacarpal joint replacement.

A specific analysis of the dual-mobility Moovis prosthesis with a total of 265 implants in 3 studies reported a failure rate of 2.6% and cup loosening of 0.4% after a mean follow-up of 48 months, which is remarkably lower compared to the single-mobility implants (e.g. ARPE 4.8% loosening, 6.8% dislocation, IVORY 1.5% loosening, 4.4% dislocation) [17, 32–34]. In our study, 1 revision with a neck exchange was performed due to restricted movement, but none of the patients had signs of loosening or dislocation. In retrospect, the reason for the limited mobility was most likely the retained volar beak at the base of the first metacarpal. In current literature, the resection of the beak is clearly recommended to avoid impingement and instability as well as facilitate exposure [35]. Shrinkage of the joint capsule is now recognized as a common cause of impaired postoperative mobility. Primary capsular release is therefore recommended [36]. As the cases included in this study were operated before this publication, this procedure was not performed in either the Elektra or the Moovis group.

Dremstrup et al. published the 2-year-results of 200 Moovis prosthesis in 2020, revealing a cumulative survival of 97%, comparable to the survival of 95.2% that we detected in our cohort after the mean follow-up of 41.4 months [37]. In addition, reoperations were performed twice in the Moovis cohort due to de Quervains disease after 5 and 9 months, which is not performed regularly anymore due to the oftentimes self-limiting course of the disease. Gonzalez-Espino et al. recommend releasing the abductor pollicis longus and extensor pollicis brevis during joint replacement surgery to prevent the development of de Quervains disease [38]. This is in accordance with a study that reported an incidence of 17% of de Quervain tendinopathy during the first year after joint replacement [4].

Long-term studies comparing trapeziometacarpal total joint replacement with resection arthroplasty are currently pending. However, the prostheses are still associated with a higher rate of adverse events leading to revision due to implant loosening or dislocation compared to trapeziectomy [39–42]. As trapeziectomy is an irreversible procedure, the options for salvage surgery after failure remain limited. In the management of adverse events after total joint arthroplasty, secondary trapeziectomy with capsule closure is currently the gold standard for the management of adverse events and can be performed with comparable results to primary resection arthroplasty [10–12]. Other salvage options after failed TMC arthroplasty can be discussed based on individual risk profiles and patient characteristics, in particular reconstructive exchange procedures to preserve the endoprosthesis [43].

As previously reported, TMC joint replacement can efficiently correct MCP1 hyperextension. In all of the 6 patients in our study, hyperextension could be corrected postoperatively. This is an important difference to common trapeziectomy, as z-deformity is usually not addressed and needs to be corrected with additional measures (e.g. MCP1 capsulodesis) in order to restore the physiological joint position [44, 45]. In studies comparing resection arthroplasty to single-mobility prostheses, patient-reported outcomes, range of motion and grip strength after prosthetic replacement were mostly superior, and recovery was oftentimes quicker [5, 7, 8, 46–49]. After all, secondary trapeziectomy remains as a salvage procedure after implant failure with comparably good outcomes regarding primary trapeziectomy [10, 12, 50].

Concerning our clinical results, we compared the DASH scores of healthy individuals to the ones that we observed after the intervention. Jester et al. published a study in 2015 on the hand function of the working population in Germany, which revealed a mean DASH score in the age group of 50 to 65 years of 19.0 +/- 18 [51]. Similarly, Aasheim and Finsen calculated a mean DASH of 18 in women age 60 to 69 in Norway [52]. With a mean patient age of 64 years at surgery in our study and a mean DASH of 25.9 in the Moovis and 20.6 in the Elektra group, these data appear comparable to the observed healthy populations, indicating satisfactory restoration of the age-related hand function after surgery. Other studies focusing on postoperative hand function after TMC joint replacement with the Moovis implant showed postoperative DASH scores ranging from 12 to 35 after surgery as well as MHQ scores of 87 to 90%, which is overall comparable to our findings [32–34]. While in literature, the Elektra implant performed worse in terms of patient-reported outcomes with DASH scores between 23 and 38 than our cohort (20.6), the low patient number in our study needs to be highlighted as a limitation [53–55].

As demonstrated, our results of range of motion and strength after surgery were comparable to the contralateral, not affected hand. One of the few studies with a long-term follow-up of at least 10 years after implantation of the Ivory prosthesis confirmed that the pinch strength was still comparable to the contralateral hand [13]. However, it must be considered that hand function of the non-operated hand might also be restricted due to secondary conditions such as osteoarthritis of the wrist or carpal tunnel syndrome. It needs to be stated that the mean postoperative grip and pinch strength determined in our study appear to be lower than in other studies: in the systematic review by Holme et al., the Elektra implants lead to grip strengths of 23 to 28 kg and pinch strengths of 3.6 to 7 kg compared to 18.9 kg (11.0-26.8) and 3.6 kg (3.0-4.1) in our Elektra group, while the Moovis implants achieved grip strengths of 21 to 28 kg and pinch strengths of 7 to 7.5 kg compared to 19.0 (14.4–23.5) and 3.4 (3.1–3.7) in our Moovis group [17]. As the non-operated hands achieved similar results, operator errors as well as miscalibration of the tools are possible reasons. On the other hand, it might be attributed to the rather small number of patients as indicated by the large confidence intervals.

Range of motion regarding the Kapandji score (literature: 8.2–10, our study: Moovis 9.6, Elektra 9.6), palmar abduction (literature: 40–50°, our study: Moovis 47.5°, Elektra 45.9°) and radial abduction (literature: 45–56°, our study: Moovis 52.2°, Elektra 53.6°) show at least comparably good results to previous literature with regards to Elektra or Moovis implants [17, 20, 32–34, 42, 54, 56].

Satisfaction was high in our study as 86% (Moovis) to 89% (Electra) were at least satisfied with the result. A total of 96% of patients would recommend the treatment and choose the surgery again. These results are comparable to earlier studies with satisfaction rates of 84.5 to 98% [13, 26, 34, 56, 57]. In another study comparing joint replacement to trapeziectomy, the ARPE prosthesis was recommended by 89% of patients vs. 76% in the trapeziectomy group [46] – despite the higher rate of revision surgery due to prosthesis failure.

### Limitations

As the presented data was analyzed retrospectively, preoperative clinical data was not available for comparison. Moreover, follow-up was not standardized to a certain time point, which is why the follow-up period differs widely. As the surgery was performed by one single surgeon over the course of time, factors such as the individual learning curve need to be considered and may alter the findings depending on the timepoint of surgery. On the other hand, it guarantees a high homogeneity and ensures that all patients are operated with the same technique and under comparable circumstances. As a rather small number of patients were operated over a long period of time of 10 years, a selection bias in the choice of patients can be assumed. Current literature highlights the importance of the surgical technique and the general difficulty of the surgery, but also concludes promising prosthesis survival rates of > 90% after 10 years with metal-on-PE bearings [35]. Due to low patient numbers especially in the Elektra group, only descriptive statistics could be used to compare the two prosthesis models in our study.

## Conclusion

In this mid- to long-term retrospective analysis, total joint replacement in primary trapeziometacarpal joint arthritis results in low pain levels, good mobility and good clinical function. Patient satisfaction is overall high. While revision due to cup loosening occurred only in patients with single-mobility implants, no cases of dislocation or loosening of components were observed in the dual-mobility group. The dual mobility TMC prosthesis seems to be a promising and reliable option for treatment of TMC joint arthritis and might be able to take over the position of the first-line therapy in the future.

## Data Availability

The datasets used and/or analyzed during the current study are available from the corresponding author on reasonable request.

## Abbreviations

CMC1: first carpometacarpal
TMC: trapeziometacarpal
VAS: visual analogue scale
CI: confidence interval
MCP1: first metacarpophalangeal
MHQ: Michigan Hand Questionnaire
DASH: Disabilities of the Arm, Shoulder and Hand questionnaire

## References

[1] Gervis WH (1949). Excision of the trapezium for osteoarthritis of the trapezio-metacarpal joint. J Bone Joint Surg Br.

[2] Vadstrup LS, Schou L, Boeckstyns ME (2009). Basal joint osteoarthritis of the thumb treated with Weilby arthroplasty: a prospective study on the early postoperative course of 106 consecutive cases. J Hand Surg Eur Vol.

[3] Degeorge B, Dagneaux L, Andrin J, Lazerges C, Coulet B, Chammas M (2018). Do trapeziometacarpal prosthesis provide better metacarpophalangeal stability than trapeziectomy and ligamentoplasty?. Orthop Traumatol Surg Res.

[4] Goubau JF, Goubau L, Goorens CK, van Hoonacker P, Kerckhove D, Vanmierlo B (2015). De Quervain Tenosynovitis following Trapeziometacarpal Ball-and-socket joint replacement. J Wrist Surg.

[5] Robles-Molina MJ, Lopez-Caba F, Gomez-Sanchez RC, Cardenas-Grande E, Pajares-Lopez M, Hernandez-Cortes P (2017). Trapeziectomy with Ligament Reconstruction and Tendon Interposition Versus a Trapeziometacarpal Prosthesis for the treatment of Thumb basal joint osteoarthritis. Orthopedics.

[6] Jager T, Barbary S, Dap F, Dautel G (2013). [Evaluation of postoperative pain and early functional results in the treatment of carpometacarpal joint arthritis. Comparative prospective study of trapeziectomy vs. MAIA((R)) prosthesis in 74 female patients]. Chir Main.

[7] Ulrich-Vinther M, Puggaard H, Lange B (2008). Prospective 1-year follow-up study comparing joint prosthesis with tendon interposition arthroplasty in treatment of trapeziometacarpal osteoarthritis. J Hand Surg Am.

[8] Cebrian-Gomez R, Lizaur-Utrilla A, Sebastia-Forcada E, Lopez-Prats FA (2019). Outcomes of cementless joint prosthesis versus tendon interposition for trapeziometacarpal osteoarthritis: a prospective study. J Hand Surg Eur Vol.

[9] Wolf JM, Atroshi I, Zhou C, Karlsson J, Englund M (2018). Sick leave after surgery for Thumb Carpometacarpal Osteoarthritis: a Population-based study. J Hand Surg Am.

[10] Kaszap B, Daecke W, Jung M (2013). Outcome comparison of primary trapeziectomy versus secondary trapeziectomy following failed total trapeziometacarpal joint replacement. J Hand Surg Am.

[11] Hansen TB, Homilius M (2010). Failed total carpometacarpal joint prosthesis of the thumb: results after resection arthroplasty. Scand J Plast Reconstr Surg Hand Surg.

[12] Cerlier A, Guinard D, Gay AM, Legre R (2021). Outcomes of secondary trapeziectomy after revision of trapeziometacarpal implants: a retrospective comparative matched study. J Hand Surg Eur Vol.

[13] Tchurukdichian A, Guillier D, Moris V, See LA, Macheboeuf Y (2020). Results of 110 IVORY(R) prostheses for trapeziometacarpal osteoarthritis with a minimum follow-up of 10 years. J Hand Surg Eur Vol.

[14] Martin-Ferrero M, Simon-Perez C, Coco-Martin MB, Vega-Castrillo A, Aguado-Hernandez H, Mayo-Iscar A (2020). Trapeziometacarpal total joint arthroplasty for osteoarthritis: 199 patients with a minimum of 10 years follow-up. J Hand Surg Eur Vol.

[15] Christiansen T (1969). A new hip prosthesis with trunnion-bearing. Acta Chir Scand.

[16] Terrier A, Latypova A, Guillemin M, Parvex V, Guyen O (2017). Dual mobility cups provide biomechanical advantages in situations at risk for dislocation: a finite element analysis. Int Orthop.

[17] Holme TJ, Karbowiak M, Clements J, Sharma R, Craik J, Ellahee N (2021). Thumb CMCJ prosthetic total joint replacement: a systematic review. EFORT Open Rev.

[18] Schmidt I (2015). Surgical Treatment options in Thumb Carpometacarpal Osteoarthritis: a recent literature overview searching for Practice Pattern with Special Focus on total joint replacement. Curr Rheumatol Rev.

[19] Eaton RG, Lane LB, Littler JW, Keyser JJ (1984). Ligament reconstruction for the painful thumb carpometacarpal joint: a long-term assessment. J Hand Surg Am.

[20] Regnard PJ (2006). Electra Trapezio metacarpal prosthesis: results of the first 100 cases. J Hand Surg Br.

[21] Berger AJ, Momeni A, Ladd AL (2014). Intra- and interobserver reliability of the Eaton classification for trapeziometacarpal arthritis: a systematic review. Clin Orthop Relat Res.

[22] Becker SJ, Teunis T, Ring D, Vranceanu AM (2016). The Trapeziometacarpal arthrosis symptoms and disability questionnaire: development and preliminary validation. Hand (N Y).

[23] Ritchie JF, Belcher HJ (2008). A comparison of trapeziectomy via anterior and posterior approaches. J Hand Surg Eur Vol.

[24] Baek Hansen T (2021). Joint replacement for trapeziometacarpal osteoarthritis: implants and outcomes. J Hand Surg Eur Vol.

[25] Mangan F, Spece H, Weiss AC, Ladd AL, Stockmans F, Kurtz SM. A review of wear debris in thumb base joint implants. Eur J Orthop Surg Traumatol. 2023. 10.1007/s00590-023-03622-x 37439887

[26] Lussiez B, Falaise C, Ledoux P (2021). Dual mobility trapeziometacarpal prosthesis: a prospective study of 107 cases with a follow-up of more than 3 years. J Hand Surg Eur Vol.

[27] Duerinckx J, Caekebeke P (2016). Trapezium anatomy as a radiographic reference for optimal cup orientation in total trapeziometacarpal joint arthroplasty. J Hand Surg Eur Vol.

[28] Caekebeke P, Duerinckx J (2018). Can surgical guidelines minimize complications after Maia(R) trapeziometacarpal joint arthroplasty with unconstrained cups?. J Hand Surg Eur Vol.

[29] Lajoinie L, Barbary S (2023). Total trapeziometacarpal prosthesis: radio-clinical advice on cup implantation. Hand Surg Rehabil.

[30] Schmidt I (2018). Treatment Options for Thumb Carpometacarpal Joint Osteoarthritis with an update to the ArpeTM Prosthesis. Recent Adv Arthroplasty.

[31] Druel T, Cievet-Bonfils M, Comtet JJ, Gazarian A. 31 years survival rate of ARPE(R) single-mobility prosthesis in trapeziometacarpal osteoarthritis. J Hand Surg Eur Vol. 2023:17531934231221692. 10.1177/17531934231221692 38114074

[32] Tchurukdichian A, Gerenton B, Moris V, See LA, Stivala A, Guillier D (2021). Outcomes of double-mobility prosthesis in Trapeziometacarpal Joint Arthritis with a minimal 3 years of Follow-Up: an advantage for Implant Stability. Hand (N Y).

[33] Dreant N, Poumellec MA (2019). Total Thumb Carpometacarpal Joint Arthroplasty: a retrospective functional study of 28 MOOVIS prostheses. Hand (N Y).

[34] Martins A, Charbonnel S, Lecomte F, Athlani L (2020). The moovis(R) implant for trapeziometacarpal osteoarthritis: results after 2 to 6 years. J Hand Surg Eur Vol.

[35] Duerinckx J, Verstreken F (2022). Total joint replacement for osteoarthritis of the carpometacarpal joint of the thumb: why and how?. EFORT Open Rev.

[36] Van Hove B, Vantilt J, Bruijnes A, Caekebeke P, Corten K, Degreef I (2020). Trapeziometacarpal total joint arthroplasty: the effect of capsular release on range of motion. Hand Surg Rehabil.

[37] Dremstrup L, Thillemann JK, Kirkeby L, Larsen LP, Hansen TB, Stilling M (2021). Two-year results of the moovis trapeziometacarpal joint arthroplasty with focus on early complications. J Hand Surg Eur Vol.

[38] Gonzalez-Espino P, Pottier M, Detrembleur C, Goffin D (2021). Touch(R) double mobility arthroplasty for trapeziometacarpal osteoarthritis: outcomes for 92 prostheses. Hand Surg Rehabil.

[39] Cooney WP, Leddy TP, Larson DR (2006). Revision of thumb trapeziometacarpal arthroplasty. J Hand Surg Am.

[40] Sandvall BK, Cameron TE, Netscher DT, Epstein MJ, Staines KG, Petersen NJ (2010). Basal joint osteoarthritis of the thumb: ligament reconstruction and tendon interposition versus hematoma distraction arthroplasty. J Hand Surg Am.

[41] Gangopadhyay S, McKenna H, Burke FD, Davis TR (2012). Five- to 18-year follow-up for treatment of trapeziometacarpal osteoarthritis: a prospective comparison of excision, tendon interposition, and ligament reconstruction and tendon interposition. J Hand Surg Am.

[42] Thorkildsen RD, Rokkum M (2019). Trapeziectomy with LRTI or joint replacement for CMC1 arthritis, a randomised controlled trial. J Plast Surg Hand Surg.

[43] Schmidt I (2014). Thumb CMC total exchange arthroplasty with the ARPE implant. Chir Main.

[44] Poulter RJ, Davis TR (2011). Management of hyperextension of the metacarpophalangeal joint in association with trapeziometacarpal joint osteoarthritis. J Hand Surg Eur Vol.

[45] Eaton RG, Floyd WE (1988). Thumb metacarpophalangeal capsulodesis: an adjunct procedure to basal joint arthroplasty for collapse deformity of the first ray. J Hand Surg Am.

[46] Craik JD, Glasgow S, Andren J, Sims M, Mansouri R, Sharma R (2017). Early results of the ARPE Arthroplasty Versus Trapeziectomy for the treatment of Thumb Carpometacarpal Joint Osteoarthritis. J Hand Surg Asian Pac Vol.

[47] Jurca J, Nemejc M, Havlas V (2016). [Surgical Treatment for Advanced Rhizarthrosis. Comparison of results of the Burton-Pellegrini technique and Trapeziometacarpal Joint Arthroplasty]. Acta Chir Orthop Traumatol Cech.

[48] Erne H, Scheiber C, Schmauss D, Loew S, Cerny M, Ehrl D (2018). Total endoprosthesis Versus Lundborg s Resection Arthroplasty for the treatment of Trapeziometacarpal Joint Osteoarthritis. Plast Reconstr Surg Glob Open.

[49] Martinez-Martinez F, Garcia-Hortelano S, Garcia-Panos JP, Moreno-Fernandez JM, Martin-Ferrero MA (2016). [Comparative clinical study of 2 surgical techniques for trapeziometacarpal osteoarthritis]. Rev Esp Cir Ortop Traumatol.

[50] Lenoir H, Erbland A, Lumens D, Coulet B, Chammas M (2016). Trapeziectomy and ligament reconstruction tendon interposition after failed trapeziometacarpal joint replacement. Hand Surg Rehabil.

[51] Jester A, Harth A, Germann G (2005). Measuring levels of upper-extremity disability in employed adults using the DASH Questionnaire. J Hand Surg Am.

[52] Aasheim T, Finsen V (2014). The DASH and the QuickDASH instruments. Normative values in the general population in Norway. J Hand Surg Eur Vol.

[53] Hernandez-Cortes P, Pajares-Lopez M, Robles-Molina MJ, Gomez-Sanchez R, Toledo-Romero MA, De Torres-Urrea J (2012). Two-year outcomes of Elektra prosthesis for trapeziometacarpal osteoarthritis: a longitudinal cohort study. J Hand Surg Eur Vol.

[54] Froschauer SM, Holzbauer M, Hager D, Schnelzer R, Kwasny O, Duscher D (2020). Elektra prosthesis versus resection-suspension arthroplasty for thumb carpometacarpal osteoarthritis: a long-term cohort study. J Hand Surg Eur Vol.

[55] Hansen TB, Snerum L (2008). Elektra trapeziometacarpal prosthesis for treatment of osteoarthrosis of the basal joint of the thumb. Scand J Plast Reconstr Surg Hand Surg.

[56] Chug M, Williams N, Benn D, Brindley S (2014). Outcome of uncemented trapeziometacarpal prosthesis for treatment of thumb carpometacarpal joint arthritis. Indian J Orthop.

[57] Dehl M, Chelli M, Lippmann S, Benaissa S, Rotari V, Moughabghab M (2017). Results of 115 Rubis II reverse thumb carpometacarpal joint prostheses with a mean follow-up of 10 years. J Hand Surg Eur Vol.

